# Real-world performance of indobufen versus aspirin after percutaneous coronary intervention: insights from the ASPIRATION registry

**DOI:** 10.1186/s12916-024-03374-3

**Published:** 2024-04-02

**Authors:** Chunfeng Dai, Muyin Liu, Zheng Yang, Youran Li, You Zhou, Danbo Lu, Yan Xia, Ao Chen, Chenguang Li, Hao Lu, Yuxiang Dai, Jianying Ma, Zhangwei Chen, Juying Qian, Junbo Ge

**Affiliations:** 1 Department of Cardiology, Zhongshan Hospital, Fudan University, Shanghai Institute of Cardiovascular Diseases, 180 Fenglin Road, Shanghai, 200032 China; 2 National Clinical Research Center for Interventional Medicine, 180 Fenglin Road, Shanghai, 200032 China; 3 Shanghai Clinical Research Center for Interventional Medicine, 180 Fenglin Road, Shanghai, 200032 China

**Keywords:** Indobufen, Aspirin intolerance, Percutaneous coronary intervention, Dual antiplatelet therapy

## Abstract

**Background:**

Indobufen is widely used in patients with aspirin intolerance in East Asia. The OPTION trial launched by our cardiac center examined the performance of indobufen based dual antiplatelet therapy (DAPT) after percutaneous coronary intervention (PCI). However, the vast majority of patients with acute coronary syndrome (ACS) and aspirin intolerance were excluded. We aimed to explore this question in a real-world population.

**Methods:**

Patients enrolled in the ASPIRATION registry were grouped according to the DAPT strategy that they received after PCI. The primary endpoints were major adverse cardiovascular and cerebrovascular events (MACCE) and Bleeding Academic Research Consortium (BARC) type 2, 3, or 5 bleeding. Propensity score matching (PSM) was adopted for confounder adjustment.

**Results:**

A total of 7135 patients were reviewed. After one-year follow-up, the indobufen group was associated with the same risk of MACCE versus the aspirin group after PSM (6.5% vs. 6.5%, hazard ratio [HR] = 0.99, 95% confidence interval [CI] = 0.65 to 1.52, P = 0.978). However, BARC type 2, 3, or 5 bleeding was significantly reduced (3.0% vs. 11.9%, HR = 0.24, 95% CI = 0.15 to 0.40, P < 0.001). These results were generally consistent across different subgroups including aspirin intolerance, except that indobufen appeared to increase the risk of MACCE in patients with ACS.

**Conclusions:**

Indobufen shared the same risk of MACCE but a lower risk of bleeding after PCI versus aspirin from a real-world perspective. Due to the observational nature of the current analysis, future studies are still warranted to further evaluate the efficacy of indobufen based DAPT, especially in patients with ACS.

**Trial registration:**

Chinese Clinical Trial Register (https://www.chictr.org.cn); Number: ChiCTR2300067274.

**Supplementary Information:**

The online version contains supplementary material available at 10.1186/s12916-024-03374-3.

## Condensed abstract

Indobufen is widely used in patients with aspirin intolerance in East Asia. We aimed to explore the efficacy and safety of indobufen-based dual antiplatelet therapy (DAPT) after percutaneous coronary intervention (PCI) in a real-world population using the data from the ASPIRATION registry. A total of 7135 patients were reviewed. After one-year follow-up, indobufen shared the same risk of major adverse cardiovascular and cerebrovascular events but a lower risk of bleeding versus aspirin. These results were generally consistent across different subgroups including aspirin intolerance, except that indobufen appeared to increase the risk of MACCE in patients with ACS.

## Background

Antiplatelet therapy is the key to the management of coronary heart disease. Of all the antiplatelet drugs, aspirin is considered the cornerstone. The current clinical guidelines recommend that dual antiplatelet therapy (DAPT), which refers to aspirin plus P2Y_12_ receptor antagonist, should be prescribed for patients over a period of time after percutaneous coronary intervention (PCI), which has been proven to reduce the incidence of major adverse cardiovascular and cerebrovascular events (MACCE) [1, 2]. However, it is observed that some patients cannot tolerate aspirin well [3], which seriously affects their quality of life and increases the risk of bleeding. It also impairs patients’ medication compliance, thereby increasing the subsequent incidence of ischemic events after PCI [4].

Currently, there exist some strategies to manage aspirin intolerance. In China, which owns one-fifth of the world’s population, the cardiologists tend to use indobufen, a novel cyclooxygenase inhibitor, to replace aspirin in patients with aspirin intolerance, which was already approved by the Chinese Food and Drug Administration. However, there are still few large-scale studies focused on the efficacy and safety of indobufen based DAPT. The OPTION study is so far the largest multicenter randomized controlled trial to compare the performance of indobufen versus aspirin in patients requiring DAPT, whose encouraging results have been published recently [5]. Nevertheless, this trial excluded the vast majority of patients with acute coronary syndrome (ACS) and aspirin intolerance. So the efficacy and safety of indobufen based DAPT in these patients remain unknown.

In the current study, we aimed to analyze the real-world performance of indobufen based DAPT after PCI through a large-scale registry launched by our cardiac center.

## Methods

### Data sources and study population

The current analysis was based on the data extracted from the Anti-thrombotic Strategies for Patients with aspIrin intoleRAnce after percuTaneous coronary InterventiON (ASPIRATION) registry, in which consecutive patients undergoing PCI were retrospectively enrolled from January 2020 to January 2021. The aim of this registry was to gather the real-world data on the management of aspirin intolerance after PCI in our cardiac center, which owns the largest volume of cardiac catheterization in eastern China. This registry and the current study were both approved by the local institutional review board. They were also in accordance with the Declaration of Helsinki and the STROBE statement (see Additional file 1: STROBE Checklist).

All patients enrolled in the ASPIRATION registry were assessed for eligibility, and those receiving oral anticoagulation therapy or cilostazol based DAPT were excluded. Patients were also excluded if they died during hospitalization or refused to participate in the current analysis. The remaining patients were divided into the indobufen group (indobufen 100 mg twice a day plus P2Y_12_ receptor antagonist) or aspirin group (aspirin 100 mg once a day plus P2Y_12_ receptor antagonist) according to the type of DAPT that they were prescribed at discharge.

### PCI procedure and perioperative antiplatelet therapy

The PCI procedures were performed according to the latest clinical guidelines. All patients were prescribed oral antiplatelet agents at a loading dose (generally 300 mg for aspirin, 100 to 200 mg for indobufen, 300 to 600 mg for clopidogrel, and 180 mg for ticagrelor) before the procedure. The specific techniques and strategies employed during PCI were all left to the interventional cardiologists. After PCI, generally, the cardiologists will adjust the type of DAPT according to the complaints and laboratory test results from the patients, since there are a significant number of patients who cannot tolerate aspirin well in China. Aspirin intolerance was defined as any conditions that prevent patients from long-term use of low-dose aspirin, such as having contraindications (e.g., peptic ulcer, gout) or severe adverse drug reactions (e.g., gastrointestinal symptoms, bleeding, allergic reactions) after taking it [3]. If this is the case, indobufen or cilostazol may be used as an alternative, depending on the patient’s preference and clinical situation. The duration of DAPT after PCI varies according to the risk of ischemia and bleeding, generally ranging from 6 to 12 months.

### Follow-up and study endpoints

After discharge, patients were followed up until occurrence of a study endpoint of interest, or up to one year, whichever came first. The study endpoints were recorded through an electronic medical record system, or telephone interview when necessary. The follow-up was completed by five trained research assistants in our cardiac center. And an independent clinical events committee blinded to the group assignment adjudicated all clinical events.

The primary efficacy endpoint was MACCE, which referred to a composite of cardiovascular death, nonfatal myocardial infarction, clinically driven repeated revascularization, definite or probable stent thrombosis, and nonfatal ischemic stroke. The primary safety endpoint was Bleeding Academic Research Consortium (BARC) defined type 2, 3, or 5 bleeding [6]. The secondary endpoints included: 1) a composite of cardiovascular death, nonfatal myocardial infarction, definite or probable stent thrombosis, and nonfatal ischemic stroke; 2) all-cause death; 3) the individual components of MACCE; 4) net adverse clinical events (NACE) defined as a composite of MACCE and BARC type 2, 3, or 5 bleeding; 5) BARC defined bleeding; 6) Thrombolysis in Myocardial Infarction (TIMI) defined bleeding [7]; 7) Global Utilization of Streptokinase and Tissue plasminogen activator for Occluded coronary arteries (GUSTO) defined bleeding [8].

### Statistical analysis

Continuous variables are expressed as mean (standard deviation) or median (interquartile range) according to the distributions examined by the quantile–quantile (Q-Q) plots. The Student’s *t*-test or the Mann–Whitney test was used to make comparisons, respectively. Categorical variables are expressed as numbers (percentage) and were compared through the Chi-square test or the Fisher’s exact test, as appropriate. The Kaplan–Meier method was used to analyze the time-to-event data, and the log-rank test was carried out to make comparisons. The difference between groups was also evaluated by the absolute standardized difference (ASD), which was more recommended in propensity score matching (PSM) [9]. An ASD > 10% was considered a meaningful difference.

The effect of different groups on endpoints was first evaluated by the univariate Cox proportional risk regression model, which yielded the unadjusted hazard ratio (HR) and 95% confidence interval (CI). Additionally, PSM was used to adjust for the confounding factors [10] (see details in Additional file 1: Supplemental Methods), which produced the matched cohort. In the matched cohort, the HR and 95% CI was also calculated by the univariate Cox proportional risk regression model with a robust sandwich variance estimator to account for the matched design. The covariate balance achieved by PSM was assessed by calculating the ASD between groups and drawing the Love plot.

Several sensitivity analyses were done. First, other statistical approaches aiming to adjust for the confounding effect (i.e., traditional multivariate regression analysis, propensity score adjustment analysis, inverse probability of treatment weighting analysis) were performed (see details in Additional file 1: Supplemental Methods). Second, patients lost to follow-up were excluded. Third, patients with unplanned drug discontinuation were excluded. The unplanned drug discontinuation was defined as the discontinuation of indobufen or aspirin due to adverse drug reactions, cost reasons, poor compliance, or anticoagulation requirement, rather than converting to single antiplatelet therapy as approved by the cardiologists. The heterogeneity of exposure effect on primary endpoints was further examined in subgroup analysis.

There was no missingness for all variables listed in Table 1, except for body mass index. The missing data were imputed to the overall median value since the percentage of missingness was fairly low (< 0.1%).

**Table 1:** Comparison of baseline characteristics before and after propensity score matching

	Before matching				After matching			
	Indobufen group (*n* = 689)	Aspirin group (*n* = 6446)	*P*	ASD	Indobufen group (*n* = 662)	Aspirin group (*n* = 662)	*P*	ASD
Age, yrs	66.3 (9.5)	63.2 (10.6)	< 0.001	0.306	66.0 (9.4)	66.0 (10.5)	0.991	0.001
Gender			< 0.001	0.157			0.626	0.030
Male	495 (71.8)	5066 (78.6)			478 (72.2)	469 (70.8)		
Female	194 (28.2)	1380 (21.4)			184 (27.8)	193 (29.2)		
BMI, kg/m^2^	24.8 (3.3)	25.0 (3.4)	0.105	0.066	24.8 (3.3)	24.8 (3.5)	0.806	0.014
Hypertension	456 (66.2)	4162 (64.6)	0.423	0.034	437 (66.0)	424 (64.0)	0.489	0.041
Diabetes	231 (33.5)	2109 (32.7)	0.699	0.017	221 (33.4)	222 (33.5)	1.000	0.003
Hyperlipidemia	166 (24.1)	1538 (23.9)	0.929	0.005	160 (24.2)	152 (23.0)	0.650	0.028
Current smoker	119 (17.3)	1261 (19.6)	0.163	0.059	117 (17.7)	109 (16.5)	0.609	0.032
Previous MI	91 (13.2)	1067 (16.6)	0.027	0.094	89 (13.4)	91 (13.7)	0.936	0.009
Previous stroke	54 (7.8)	401 (6.2)	0.117	0.063	51 (7.7)	44 (6.6)	0.523	0.041
Previous PCI	172 (25.0)	1956 (30.3)	0.004	0.121	168 (25.4)	160 (24.2)	0.656	0.028
Previous CABG	8 (1.2)	85 (1.3)	0.865	0.014	8 (1.2)	8 (1.2)	1.000	< 0.001
CKD	100 (14.5)	631 (9.8)	< 0.001	0.145	95 (14.4)	92 (13.9)	0.875	0.013
Gastrointestinal diseases	251 (36.4)	285 (4.4)	< 0.001	0.865	224 (33.8)	214 (32.3)	0.599	0.032
ARC-HBR	186 (27.0)	1094 (17.0)	< 0.001	0.244	175 (26.4)	198 (29.9)	0.179	0.077
SBP, mmHg	133.3 (18.0)	132.9 (19.0)	0.665	0.018	133.2 (17.9)	133.5 (19.6)	0.782	0.015
DBP, mmHg	77.7 (11.4)	78.3 (10.9)	0.181	0.053	77.6 (11.4)	78.1 (11.3)	0.506	0.037
Heart rate, bpm	75.2 (11.4)	75.0 (11.3)	0.574	0.022	75.2 (11.5)	75.6 (11.3)	0.478	0.039
Diagnosis at admission			0.104	0.098			0.787	0.057
Stable angina	398 (57.8)	3854 (59.8)			385 (58.2)	395 (59.7)		
Unstable angina	191 (27.7)	1528 (23.7)			179 (27.0)	165 (24.9)		
NSTEMI	65 (9.4)	703 (10.9)			65 (9.8)	64 (9.7)		
STEMI	35 (5.1)	361 (5.6)			33 (5.0)	38 (5.7)		
ACS	291 (42.2)	2592 (40.2)			277 (41.8)	267 (40.3)		
Primary PCI	28 (4.1)	260 (4.0)	1.000	0.002	26 (3.9)	28 (4.2)	0.889	0.015
Radial artery access	676 (98.1)	6320 (98.0)	1.000	0.005	13 (2.0)	11 (1.7)	0.837	0.023
Multivessel disease	467 (67.8)	4610 (71.5)	0.044	0.081	453 (68.4)	452 (68.3)	1.000	0.003
Target vessel			0.787	0.059			0.957	0.057
Left main	4 (0.6)	40 (0.6)			4 (0.6)	4 (0.6)		
Left anterior descending	274 (39.7)	2425 (37.6)			263 (39.7)	261 (39.4)		
Left circumflex	82 (11.9)	781 (12.1)			80 (12.1)	83 (12.5)		
Right coronary artery	152 (22.1)	1524 (23.7)			142 (21.5)	154 (23.3)		
Grafts	2 (0.3)	9 (0.1)			2 (0.3)	2 (0.3)		
Multiple	175 (25.4)	1667 (25.9)			171 (25.8)	158 (23.9)		
Lesion characteristics								
Burfication	110 (16.0)	993 (15.4)	0.740	0.015	105 (15.9)	102 (15.4)	0.880	0.012
Calcified	84 (12.2)	643 (10.0)	0.078	0.071	79 (11.9)	85 (12.8)	0.677	0.028
In-stent restenosis	45 (6.5)	486 (7.5)	0.378	0.039	43 (6.5)	27 (4.1)	0.065	0.108
Chronic total occlusion	77 (11.2)	891 (13.8)	0.061	0.080	75 (11.3)	75 (11.3)	1.000	< 0.001
PCI strategy			0.826	0.025			0.818	0.035
PTCA	9 (1.3)	100 (1.6)			9 (1.4)	10 (1.5)		
Stent implantation	620 (90.0)	5812 (90.2)			595 (89.9)	600 (90.6)		
Drug-coated balloon	60 (8.7)	534 (8.3)			58 (8.8)	52 (7.9)		
Intravenous GPI	82 (11.9)	1005 (15.6)	0.012	0.107	79 (11.9)	81 (12.2)	0.933	0.009
LVEF, %	61.3 (7.6)	60.6 (7.7)	0.024	0.091	61.2 (7.7)	61.4 (7.0)	0.579	0.031
Hemoglobin, g/L	130.0 (16.9)	134.0 (15.4)	< 0.001	0.246	130.1 (17.0)	129.6 (16.3)	0.584	0.030
Platelet, × 10^9^/L	189.0 (158.0, 232.0)	191.0 (157.0, 235.0)	0.617	0.021	189.0 (158.0, 233.0)	189.0 (159.0, 239.0)	0.607	0.035
Serum creatinine, μmol/L	82.0 (71.0, 94.0)	81.0 (70.0, 92.0)	0.044	0.060	82.0 (71.0, 94.0)	81.0 (69.0, 93.0)	0.188	0.009
Positive FOBT	152 (22.1)	718 (11.1)	< 0.001	0.297	142 (21.5)	140 (21.1)	0.946	0.007
P2Y_12_ receptor antagonist			< 0.001	0.272			1.000	< 0.001
Clopidogrel	536 (77.8)	4231 (65.6)			510 (77.0)	510 (77.0)		
Ticagrelor	153 (22.2)	2215 (34.4)			152 (23.0)	152 (23.0)		
Statin	676 (98.1)	6354 (98.6)	0.432	0.036	652 (98.5)	649 (98.0)	0.674	0.035
Ezetimibe	102 (14.8)	910 (14.1)	0.665	0.020	98 (14.8)	103 (15.6)	0.759	0.021
Fenofibrate	9 (1.3)	83 (1.3)	1.000	0.002	9 (1.4)	6 (0.9)	0.604	0.043
PCSK9i	6 (0.9)	98 (1.5)	0.236	0.060	6 (0.9)	2 (0.3)	0.287	0.078
RASI	365 (53.0)	3555 (55.2)	0.294	0.044	352 (53.2)	336 (50.8)	0.409	0.048
ARNI	35 (5.1)	440 (6.8)	0.095	0.074	35 (5.3)	29 (4.4)	0.522	0.042
Βeta-blocker	458 (66.5)	4552 (70.6)	0.027	0.089	440 (66.5)	442 (66.8)	0.954	0.006
Ivabradine	9 (1.3)	74 (1.1)	0.856	0.014	9 (1.4)	7 (1.1)	0.801	0.028
CCB	226 (32.8)	1907 (29.6)	0.087	0.069	214 (32.3)	222 (33.5)	0.682	0.026
Nitrate	222 (32.2)	2131 (33.1)	0.687	0.018	205 (31.0)	210 (31.7)	0.813	0.016
Diuretic	53 (7.7)	490 (7.6)	0.992	0.003	50 (7.6)	53 (8.0)	0.837	0.017
PPI	482 (70.0)	2606 (40.4)	< 0.001	0.622	458 (69.2)	460 (69.5)	0.952	0.007

Values are shown as numbers (%), mean (standard deviation), or median (interquartile range)

*Abbreviations*: *ACS* acute coronary syndrome, *ARC-HBR* Academic Research Consortium-high bleeding risk, *ARNI* angiotensin receptor-neprilysin inhibitor, *ASD* absolute standardized difference, *BMI* body mass index, *CABG* coronary artery bypass grafting, *CCB* calcium channel blocker, *CKD* chronic kidney disease, *DBP* diastolic blood pressure, *FOBT* fecal occult blood test, *GPI* glycoprotein IIb/IIIa receptor inhibitor, *LVEF* left ventricular ejection fraction, *MI* myocardial infarction, *NSTEMI* non-ST-segment elevation myocardial infarction, *PCI* percutaneous coronary intervention, *PCSK9i* proprotein convertase subtilisin/kexin type 9 inhibitor, *PPI* proton pump inhibitor, *PTCA* percutaneous transluminal coronary angioplasty, *RASI* renin-angiotensin system inhibitor, *SBP* systolic blood pressure, *STEMI* ST-segment elevation myocardial infarction

The statistical analysis was performed using *IBM SPSS Statistics* software version 25.0 (IBM Corp., Armonk, New York, USA) and *R* software version 4.0.0 (*R* Foundation for Statistical Computing, Vienna, Austria). A two-tailed *p* value < 0.05 was considered statistically significant.

## Results

### Population and matching

**Fig. 1:**
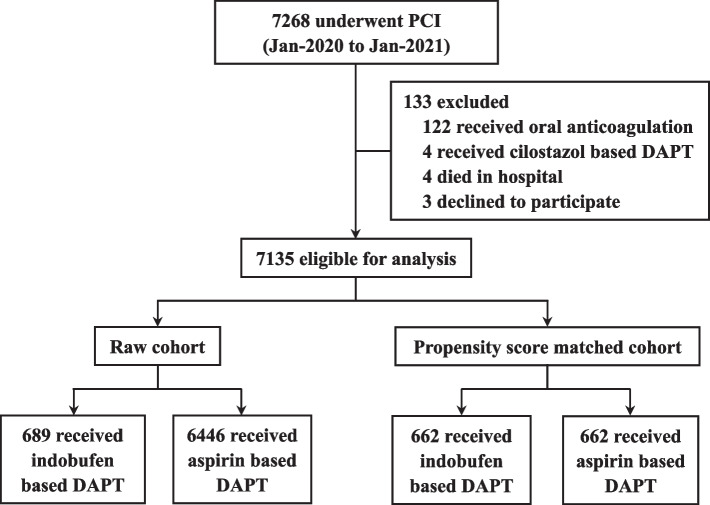
Study flow chart. Abbreviations: DAPT = dual antiplatelet therapy; PCI = percutaneous coronary intervention

As shown in Fig. 1, a total of 7268 patients undergoing PCI were assessed for eligibility, and 133 were excluded. Among the remaining 7135 patients, the mean age was 63.5 ± 10.5 years and 5561 (77.9%) were male. At discharge, 689 (9.7%) patients were prescribed indobufen based DAPT due to aspirin intolerance, and the use of indobufen increased gradually in our center (Additional file 1: Figure S1). Gastrointestinal intolerance, including gastrointestinal bleeding, digestive discomfort, or pre-existing digestive diseases (such as ulcers), was the major manifestations of aspirin intolerance (Additional file 1: Figure S2).

The baseline characteristics are shown in Table 1. Before matching, there were significant differences between the two groups. Patients in the indobufen group tended to have risk factors for bleeding like advanced age, female gender, chronic kidney disease, and gastrointestinal disease. Lower levels of hemoglobin and higher rates of positive fecal occult blood test were also more common in the indobufen group. In terms of medications at discharge, patients in the indobufen group were more likely to be prescribed clopidogrel and proton pump inhibitor (PPI). The two groups were comparable on angiographic and procedural characteristics.

After 1:1 PSM, there were 662 patients in both groups, and all baseline characteristics were well-balanced (Table 1, Additional file 1: Figure S3). The distributions of propensity scores before and after matching are shown in Additional file 1: Figure S4.

### Primary endpoints

**Fig. 2:**
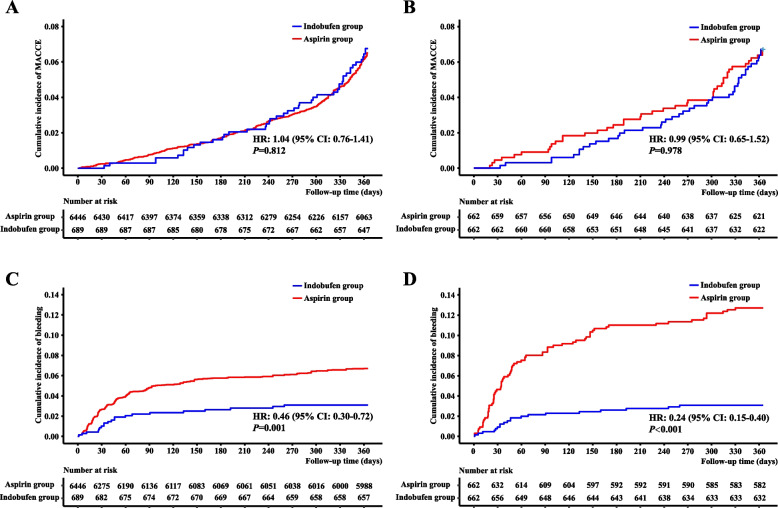
Cumulative incidence of primary endpoints. The top two panels show the results of MACCE for both groups in the raw (A, log-rank *P* = 0.812) and propensity score matched cohort (B, log-rank *P* = 0.978), while the bottom two panels show the results of BARC type 2, 3, or 5 bleeding in the raw (C, log-rank *P* < 0.001) and propensity score matched cohort (D, log-rank *P* < 0.001). Abbreviations: BARC = Bleeding Academic Research Consortium; CI = confidence interval; HR = hazard ratio; MACCE = major adverse cardiovascular and cerebrovascular events

**Fig. 3:**
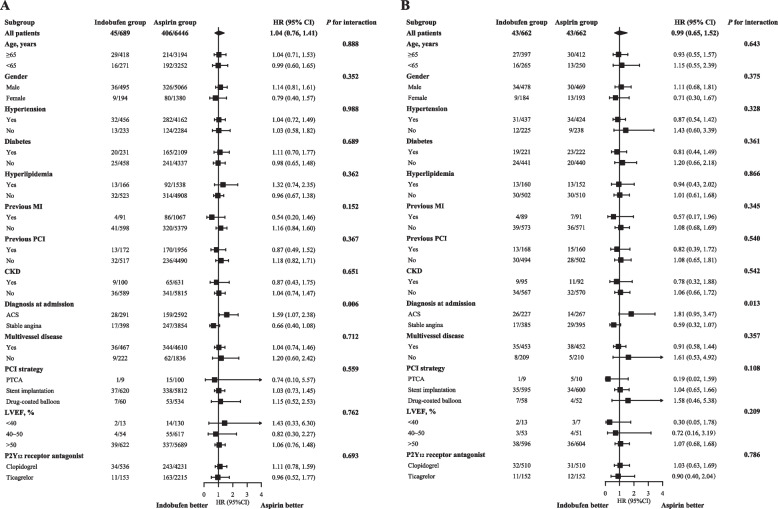
Subgroup analysis for MACCE. It shows the subgroup analysis for MACCE before (A) and after (B) propensity score matching. Abbreviations: ACS = acute coronary syndrome; CI = confidence interval; CKD = chronic kidney disease; HR = hazard ratio; LVEF = left ventricular ejection fraction; MACCE = major adverse cardiovascular and cerebrovascular events; MI = myocardial infarction; PCI = percutaneous coronary intervention; PTCA = percutaneous transluminal coronary angioplasty

During one-year follow-up, the two groups had similar risk of MACCE both in the raw (6.5% vs. 6.3%, unadjusted HR: 1.04, 95% CI: 0.76 to 1.42, *P* = 0.812) and propensity score matched (6.5% vs. 6.5%, adjusted HR: 0.99, 95% CI: 0.65 to 1.52, *P* = 0.978) cohort (Table 2). The cumulative incidence curves of MACCE are presented in Fig. 2. The above results were consistent across the prespecified subgroups except for diagnosis at admission (Fig. 3). The use of indobufen appeared to increase the risk of MACCE in patients with ACS.

**Table 2:** Primary and secondary endpoints

	Raw cohort (*n* = 7135)				Propensity score matched cohort (*n* = 1324)			
	Indobufen group (*n* = 689)	Aspirin group (*n* = 6446)	HR (95% CI)	*P*	Indobufen group (*n* = 662)	Aspirin group (*n* = 662)	HR (95% CI)	*P*
Primary endpoints								
MACCE	45 (6.5%)	406 (6.3%)	1.04 (0.76, 1.41)	0.812	43 (6.5%)	43 (6.5%)	0.99 (0.65, 1.52)	0.978
BARC type 2, 3, or 5 bleeding	21 (3.0%)	418 (6.5%)	0.46 (0.30, 0.72)	0.001	20 (3.0%)	79 (11.9%)	0.24 (0.15, 0.40)	< 0.001
Secondary endpoints								
Cardiovascular death, nonfatal MI, stent thrombosis, or nonfatal ischemic stroke	19 (2.8%)	130 (2.0%)	1.37 (0.85, 2.22)	0.199	17 (2.6%)	16 (2.4%)	1.06 (0.54, 2.10)	0.862
All-cause death	11 (1.6%)	53 (0.8%)	1.95 (1.02, 3.73)	0.044	10 (1.5%)	5 (0.8%)	2.01 (0.69, 5.87)	0.204
Cardiovascular death	5 (0.7%)	26 (0.4%)	1.80 (0.69, 4.70)	0.227	4 (0.6%)	4 (0.6%)	1.00 (0.25, 4.01)	0.998
Nonfatal MI	3 (0.4%)	56 (0.9%)	0.50 (0.16, 1.60)	0.244	3 (0.5%)	7 (1.1%)	0.43 (0.11, 1.66)	0.220
Repeated revascularization	29 (4.2%)	316 (4.9%)	0.86 (0.59, 1.26)	0.435	29 (4.4%)	33 (5.0%)	0.88 (0.53, 1.44)	0.604
Stent thrombosis	2 (0.3%)	20 (0.3%)	0.94 (0.22, 4.00)	0.928	2 (0.3%)	2 (0.3%)	1.00 (0.14, 7.10)	1.000
Nonfatal ischemic stroke	4 (0.6%)	21 (0.3%)	1.79 (0.61, 5.21)	0.286	3 (0.5%)	4 (0.6%)	0.75 (0.17, 3.36)	0.707
NACE	65 (9.4%)	781 (12.1%)	0.76 (0.59, 0.98)	0.035	62 (9.4%)	113 (17.1%)	0.52 (0.38, 0.70)	< 0.001
BARC defined bleeding								
Type 1	60 (8.7%)	612 (9.5%)	0.91 (0.70, 1.19)	0.488	52 (7.9%)	106 (16.0%)	0.47 (0.34, 0.65)	< 0.001
Type 2	11 (1.6%)	266 (4.1%)	0.38 (0.21, 0.70)	0.002	10 (1.5%)	47 (7.1%)	0.21 (0.11, 0.41)	< 0.001
Type 3	10 (1.5%)	145 (2.3%)	0.64 (0.34, 1.22)	0.176	10 (1.5%)	28 (4.2%)	0.35 (0.17, 0.73)	0.005
Type 5	0 (0.0%)	7 (0.1%)	/	0.387*	0 (0.0%)	4 (0.6%)	/	0.045*
Minor (type 1 or 2)	71 (10.3%)	878 (13.6%)	0.74 (0.58, 0.95)	0.016	62 (9.4%)	153 (23.1%)	0.37 (0.28, 0.50)	< 0.001
Major (type 3 or 5)	10 (1.5%)	152 (2.4%)	0.61 (0.32, 1.16)	0.133	10 (1.5%)	32 (4.8%)	0.31 (0.15, 0.63)	0.001
Bleeding site								
Subcutaneous	30 (4.4%)	403 (6.3%)	0.72 (0.52, 0.99)	0.046	27 (4.1%)	64 (9.7%)	0.43 (0.30, 0.62)	< 0.001
Gastrointestinal	37 (5.4%)	500 (7.8%)	0.54 (0.39, 0.75)	0.023	33 (5.0%)	82 (12.4%)	0.34 (0.17, 0.68)	< 0.001
Urogenital	3 (0.4%)	31 (0.5%)	0.98 (0.17, 5.65)	0.868	2 (0.3%)	8 (1.2%)	0.79 (0.47, 1.33)	0.107
Intracranial	0 (0.0%)	4 (0.4%)	/	0.513*	0 (0.0%)	1 (0.2%)	/	0.317*
Other	11 (1.6%)	92 (1.4%)	0.97 (0.23, 4.10)	0.930	10 (1.5%)	30 (4.5%)	0.32 (0.14, 0.73)	0.001
TIMI defined bleeding								
Minimal	70 (10.2%)	893 (13.9%)	0.72 (0.56, 0.92)	0.008	61 (9.2%)	156 (23.6%)	0.36 (0.27, 0.48)	< 0.001
Minor	7 (1.0%)	107 (1.7%)	0.61 (0.28, 1.31)	0.206	7 (1.1%)	22 (3.3%)	0.32 (0.14, 0.74)	0.008
Major	4 (0.6%)	30 (0.5%)	1.25 (0.44, 3.54)	0.677	4 (0.6%)	7 (1.1%)	0.57 (0.17, 1.94)	0.368
GUSTO defined bleeding								
Minor	72 (10.4%)	883 (13.7%)	0.75 (0.59, 0.95)	0.018	63 (9.5%)	153 (23.1%)	0.38 (0.28, 0.51)	< 0.001
Moderate	3 (0.4%)	98 (1.5%)	0.29 (0.09, 0.90)	0.032	3 (0.5%)	21 (3.2%)	0.14 (0.04, 0.47)	0.002
Severe or life-threatening	6 (0.9%)	49 (0.8%)	1.15 (0.49, 2.67)	0.753	6 (0.9%)	11 (1.7%)	0.54 (0.20, 1.47)	0.227

^*^These* P* values were calculated by log-rank test

*Abbreviations*: *BARC* Bleeding Academic Research Consortium, *CI* confidence interval, *GUSTO* Global Utilization of Streptokinase and Tissue plasminogen activator for Occluded coronary arteries, *HR* hazard ratio, *MACCE* major adverse cardiovascular and cerebrovascular events, *MI* myocardial infarction, *NACE* net adverse clinical events, *TIMI* Thrombolysis in Myocardial Infarction

**Fig. 4:**
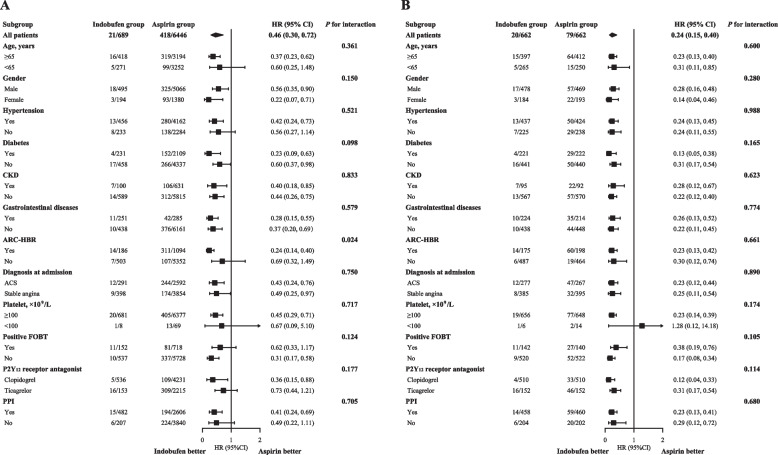
Subgroup analysis for BARC type 2, 3, or 5 bleeding. It shows the subgroup analysis for BARC type 2, 3, or 5 bleeding before (A) and after (B) propensity score matching. Abbreviations: ACS = acute coronary syndrome; ARC-HBR = Academic Research Consortium-high bleeding risk; BARC = Bleeding Academic Research Consortium; CI = confidence interval; CKD = chronic kidney disease; FOBT = fecal occult blood test; HR = hazard ratio; PPI = proton pump inhibitor

As for safety endpoint, patients in the indobufen group were less likely to suffer from BARC type 2, 3, or 5 bleeding, both in the raw (3.0% vs. 6.5%, unadjusted HR: 0.46, 95% CI: 0.30 to 0.72, *P* = 0.001) and propensity score matched (3.0% vs. 11.9%, adjusted HR: 0.24, 95% CI: 0.15 to 0.40, *P* < 0.001) cohort (Table 2). The cumulative incidence curves of BARC type 2, 3, or 5 bleeding are presented in Fig. 2. The above results were consistent across the prespecified subgroups (Fig. 4).

In addition, when other statistical approaches aiming to adjust for the confounding effect were performed, the above results were not affected (Additional file 1: Figure S5).

### Secondary endpoints

As shown in Table 2, the two groups had similar risk of other efficacy endpoints, while indobufen based DAPT tended to prevent the patients from NACE and bleeding either defined by BARC, TIMI, or GUSTO.

### Additional analyses

During one-year follow-up, there were 285 patients lost to follow-up, including 23 (3.3%) cases in the indobufen group and 262 (4.1%) cases in the aspirin group. The baseline characteristics were approximately comparable between patients who were lost and those who were not (Additional file 1: Table S1). Additionally, there were 214 patients who had unplanned drug discontinuation during follow-up, including 40 (5.8%) cases in the indobufen group and 174 (2.7%) cases in the aspirin group. The relatively high price seemed to be the major reason for indobufen discontinuation, while the adverse drug reactions still significantly affected the long-term use of aspirin (Additional file 1: Figure S6).

The additional sensitivity analyses further illustrated that the presence of loss to follow-up or unplanned drug discontinuation had limited impact on our major conclusions (Additional file 1: Figure S5).

## Discussion

To our knowledge, this study was the first real-world analysis based on a large-scale registry to evaluate the efficacy and safety of indobufen based DAPT after PCI. We observed that the indobufen group was associated with the same risk of MACCE versus the aspirin group, while the bleeding events were significantly reduced.

### Prevalence and management strategies of aspirin intolerance

Currently, there is no widely accepted definition of aspirin intolerance [3]. However, it is frequently observed that a considerable proportion of patients cannot tolerate aspirin well. In a post-hoc analysis of the SYMPHONY and 2nd SYMPHONY study, 11.9% of patients experienced gastrointestinal discomfort after taking aspirin [11]. Our previous study also found that the proportion of patients with aspirin intolerance after PCI is as high as over 10% in China [3]. In the current analysis, 689 (9.7%) patients were prescribed indobufen after PCI due to aspirin intolerance.

Aspirin intolerance can lead to decreased medication adherence. It was estimated that 9% of post-ACS patients stopped taking aspirin due to intolerance during follow-up [11]. In the current study, despite strong intervention for aspirin intolerance during hospitalization, there were still 2.7% of patients in the aspirin group stopped taking aspirin after discharge, mainly due to adverse drug reactions. Poor medication compliance will increase the incidence of ischemic events in patients requiring vigorous antiplatelet therapy, especially those taking DAPT after PCI [4].

In this regard, proton pump inhibitor (PPI) is recommended to reduce the incidence of gastrointestinal intolerance in patients requiring DAPT [12]. However, worries are emerging since long-term PPI use might be associated with the occurrence of pneumonia, bone fractures, gastric mucosa atrophy [13], lower gastrointestinal bleeding [14], or even gastrointestinal cancer [15, 16], although it is still controversial. Anyway, it is determined that the addition of PPI is not appropriate for all clinical scenarios of aspirin intolerance, especially when bleeding outside the gastrointestinal tract, allergic reactions, or gout occurs. Aspirin desensitization is another known way to deal with aspirin intolerance [17]. However, this approach has certain risks and requires interdisciplinary cooperation. It is also not practical for the mast majority of patients with ACS who need to take aspirin immediately. Therefore, there is still an urgent need for an antiplatelet drug that can replace aspirin, especially when DAPT is required.

In the past, cilostazol was widely prescribed for patients with aspirin intolerance in East Asian countries [3]. However, the use of cilostazol in Chinese patients with coronary heart disease is decreasing due to the restriction of health insurance indications. In addition, there have been concerns that cilostazol may aggravate myocardial ischemia via increasing the heart rate. It is also contraindicated in patients with heart failure.

### Indobufen and indobufen based DAPT

Indobufen, a phenylbutyrate derivative, is a relatively new generation of antiplatelet drugs, which can reversibly inhibit cyclooxygenase-1 and reduce the formation of thromboxane A2. It can also prevent the platelet aggregation induced by arachidonic acid or adenosine diphosphate, thus inhibiting the formation of thrombus [18]. Studies on healthy volunteers [19] and patients [20] showed that indobufen effectively inhibited thromboxane A2 formation, but had little effect on prostacycline, which was different from aspirin. These results theoretically indicated that indobufen had higher selectivity for platelet inhibition and better gastrointestinal tolerance, which was further confirmed by an endoscopic study [21]. In addition, unlike aspirin which irreversibly inhibits cyclooxygenase, the inhibitory effect of indobufen is transient and the platelet function can be recovered within 24 h after withdrawal [22], which significantly reduces the risk of bleeding.

At present, indobufen with a dose of 100 mg twice a day has been approved for patients with aspirin intolerance in China, and one bioequivalence study has shown that the antiplatelet effect of indobufen at this dose is comparable to that of aspirin with a dose of 100 mg once a day [23]. The clinical evidence behind such a move, however, is still scarce. Although previous studies observed that indobufen effectively prevented the ischemic events after coronary artery bypass grafting [24] and cerebral infarction [25], few studies focus on the performance of indobufen based DAPT on clinical events after PCI. The OPTION trial, initiated by our cardiac center, is considered the largest multicenter randomized controlled trial so far to compare the efficacy and safety of indobufen versus aspirin in patients requiring DAPT [5]. However, this trial only focused on patients with negative cardiac troponin, and those with aspirin intolerance were also excluded due to ethical considerations.

Therefore, the current study, based on a large-scale real-world registry, provided new clinical evidence of indobufen based DAPT from a more comprehensive perspective. We found that the indobufen group shared the same risk of MACCE with the aspirin group during one-year follow-up, which was consistent with the results from the OPTION trial [5]. A significant decrease in BARC type 2, 3, or 5 bleeding was also observed in the indobufen group. Notably, the bleeding incidence in the aspirin group (11.9%) was higher than that reported in the OPTION study (4.7%), which can be explained by the differences in the design of study and the characteristics of enrolled patients. The effect of PSM can also account for it.

The above results were generally consistent across the subgroups including aspirin intolerance. However, in patients with ACS, who were basically excluded in the OPTION trial, the use of indobufen tended to increase the risk of MACCE, although this association should be interpreted with caution owing to the nature of observational study and the limitation of subgroup analysis. Further studies are still warranted to investigate the efficacy of indobufen based DAPT in patients with ACS.

### Clinical indications

This study was originally designed to make up for the limitations of the OPTION trial and to provide a strategy from China to deal with aspirin intolerance, which is still a head-scratching problem for cardiologists around the world. This is not to say that all patients with aspirin intolerance should be prescribed with indobufen. What is important is that we should identify aspirin intolerance and help these patients choose the optimal management strategies, which include the addition of PPI, alternative drugs like indobufen or cilostazol, aspirin desensitization, and de-escalation of antiplatelet therapy. This decision ultimately relies on the specific clinical scenarios, personal willingness, and affordability.

### Limitations

Our study has some limitations. First, as an observational study, we were not able to adjust for other unmeasured confounders, although the PSM and regression analysis were adopted. Therefore, the main beneficiaries of this study are those intolerant to aspirin, rather than the entire PCI population. However, due to ethical concerns, we were not likely to design a randomized controlled trial comparing the efficacy of indobufen vs. aspirin head-to-head in patients with aspirin intolerance. Second, patients with ACS who did not receive PCI were excluded from the analysis. So our results cannot apply to these patients. Third, the proportion of ACS (especially acute myocardial infarction) was not high in this study. Therefore, future studies are still required to focus more attention on this special population. Fourth, the current study was based on single-center data and the vast majority of the evidence on indobufen came from studies performed in Asia. So the generalizability of our results should be further evaluated in other cohorts.

## Conclusions

In conclusion, indobufen based DAPT shared the same risk of MACCE but a lower risk of bleeding versus aspirin based DAPT from a real-world perspective. However, the efficacy of indobufen based DAPT, especially in patients with ACS, still need to be evaluated in future studies.

### Supplementary Information

**Additional file 1: Supplemental Methods. Table S1.** Comparison of baseline characteristics according to the status of follow-up.** Figure S1.** Temporal trend of indobufen use.** Figure S2**. Manifestations of aspirin intolerance.** Figure S3.** Comparison of ASD before and after matching.** Figure S4.** Distributions of propensity scores before and after matching.** Figure S5.** Sensitivity analysis.** Figure S6.** Reasons for unplanned drug discontinuation. **STROBE Checklist**.

## Data Availability

The data that support the findings of this study are not openly available due to reasons of sensitivity and are available from the corresponding author upon reasonable request.

## Abbreviations

ACS: Acute coronary syndrome
ASD: Absolute standardized difference
CI: Confidence interval
DAPT: Dual antiplatelet therapy
HR: Hazard ratio
LVEF: Left ventricular ejection fraction
MACCE: Major adverse cardiovascular and cerebrovascular events
PCI: Percutaneous coronary intervention
PSM: Propensity score matching
TIMI: Thrombolysis in Myocardial Infarction
