# Supplementary material for: Real-world performance of indobufen versus aspirin after percutaneous coronary intervention: insights from the ASPIRATION registry
Source: BMC Med. 2024 Apr 2;22:148. doi: 10.1186/s12916-024-03374-3 (PMC10986102; doi:10.1186/s12916-024-03374-3)
Supplement: Supplementary file 1 — Additional file 1: Supplemental Methods. Table S1. Comparison of baseline characteristics according to the status of follow-up. Figure S1. Temporal trend of indobufen use. Figure S2. Manifestations of aspirin intolerance. Figure S3. Comparison of ASD before and after matching. Figure S4. Distributions of propensity scores before and after matching. Figure S5. Sensitivity analysis. Figure S6. Reasons for unplanned drug discontinuation. STROBE Checklist. [file 12916_2024_3374_MOESM1_ESM.doc]

**SUPPLEMENTAL MATERIALS**

**Real-world performance of indobufen versus aspirin after percutaneous coronary intervention: insights from the ASPIRATION registry**

**Supplemental Methods**

***Propensity score matching***

The propensity score was calculated through a nonparsimonious logistic regression model, in which the study group was considered the dependent variable while all characteristics listed in **Table 1** were the independent variables. After ranking propensity scores in ascending order, the matching was performed using a 1:1 nearest neighbor approach without replacement with a caliper of 0.15 standard deviation of logit for propensity score, which produced the propensity score matched cohort.

***Sensitivity analysis using different statistical approaches for confounder adjustment***

In **traditional multivariate regression analysis**, the selected variables were adjusted in the Cox regression model as covariates. The variables adjusted for the occurrence of MACCE included: age, gender, body mass index, hypertension, diabetes, hyperlipidemia, current smoker, previous myocardial infarction, previous stroke, previous percutaneous coronary intervention, previous coronary artery bypass grafting, chronic kidney disease, systolic blood pressure, diastolic blood pressure, heart rate, diagnosis at admission, primary percutaneous coronary intervention, multivessel disease, target vessel, burfication lesion, calcified lesion, in-stent restenosis, chronic total occlusion, percutaneous coronary intervention strategy, left ventricular ejection fraction, P2Y_12_ receptor antagonist, statin, renin-angiotensin system inhibitor, angiotensin receptor-neprilysin inhibitor, and beta-blocker. The variables adjusted for the occurrence of BARC type 2, 3, or 5 bleeding included: age, gender, body mass index, hypertension, diabetes, hyperlipidemia, current smoker, previous myocardial infarction, previous stroke, previous percutaneous coronary intervention, previous coronary artery bypass grafting, chronic kidney disease, gastrointestinal diseases, Academic Research Consortium-high bleeding risk, systolic blood pressure, diastolic blood pressure, heart rate, diagnosis at admission, percutaneous coronary intervention strategy, hemoglobin, platelet, serum creatine, positive fecal occult blood test, P2Y_12_ receptor antagonist, and proton pump inhibitor. In **propensity score adjustment analysis**, the propensity score was considered the only covariate in the Cox regression model. In **inverse probability of treatment weighting (IPTW) analysis**, the propensity score was used to weight subjects by the inverse of treatment probability, thereby producing a pseudo-population in which between-group difference was balanced. Between-group comparisons were done using Cox regression models with use of stabilized inverse propensity score as weight.

**Supplemental Tables**

**Table S1**: Comparison of baseline characteristics according to the status of follow-up.

|  | Loss to follow-up | | *P* |
| --- | --- | --- | --- |
|  | No (*n*=6850) | Yes (*n*=285) |  |
| Age, yrs | 63.4 (10.4) | 67.9 (10.8) | <0.001 |
| Gender |  |  | 0.033 |
| Male | 5354 (78.2) | 207 (72.6) |  |
| Female | 1496 (21.8) | 78 (27.4) |  |
| BMI, kg/m^2^ | 25.0 (3.4) | 25.2 (3.4) | 0.406 |
| Hypertension | 4419 (64.5) | 199 (70.0) | 0.076 |
| Diabetes | 2236 (32.6) | 104 (36.5) | 0.196 |
| Hyperlipidemia | 1640 (23.9) | 64 (22.5) | 0.613 |
| Current smoker | 1338 (19.5) | 42 (14.7) | 0.053 |
| Previous MI | 1102 (16.1) | 56 (19.6) | 0.130 |
| Previous stroke | 430 (6.3) | 25 (8.8) | 0.118 |
| Previous PCI | 2035 (29.7) | 93 (32.6) | 0.322 |
| Previous CABG | 84 (1.2) | 9 (3.2) | 0.011 |
| CKD | 682 (10.0) | 49 (17.2) | <0.001 |
| GI problems | 520 (7.6) | 16 (5.6) | 0.260 |
| ARC-HBR | 1200 (17.5) | 80 (28.1) | <0.001 |
| SBP, mmHg | 132.9 (18.9) | 134.3 (19.0) | 0.222 |
| DBP, mmHg | 78.2 (11.0) | 77.2 (10.5) | 0.113 |
| Heart rate, bpm | 75.0 (11.2) | 75.7 (12.6) | 0.315 |
| Diagnosis at admission |  |  | 0.939 |
| Stable angina | 4081 (59.6) | 171 (60.0) |  |
| Unstable angina | 1648 (24.1) | 71 (24.9) |  |
| NSTEMI | 739 (10.8) | 29 (10.2) |  |
| STEMI | 382 (5.6) | 14 (4.9) |  |
| ACS | 2769 (40.4) | 114 (40.0) |  |
| Primary PCI | 283 (4.1) | 5 (1.8) | 0.065 |
| Radial artery access | 6718 (98.1) | 278 (97.5) | 0.678 |
| Multivessel disease | 4872 (71.1) | 205 (71.9) | 0.820 |
| Target vessel |  |  | 0.619 |
| Left main | 43 (0.6) | 1 (0.4) |  |
| Left anterior descending | 2588 (37.8) | 111 (38.9) |  |
| Left circumflex | 824 (12.0) | 39 (13.7) |  |
| Right coronary artery | 1606 (23.4) | 70 (24.6) |  |
| Grafts | 10 (0.1) | 1 (0.4) |  |
| Multiple | 1779 (26.0) | 63 (22.1) |  |
| Lesion characteristics |  |  |  |
| Burfication | 1059 (15.5) | 44 (15.4) | 1.000 |
| Calcified | 684 (10.0) | 43 (15.1) | 0.007 |
| In-stent restenosis | 507 (7.4) | 24 (8.4) | 0.598 |
| Chronic total occlusion | 926 (13.5) | 42 (14.7) | 0.617 |
| PCI strategy |  |  | 0.220 |
| PTCA | 107 (1.6) | 2 (0.7) |  |
| Stent implantation | 6167 (90.0) | 265 (93.0) |  |
| Drug-coated balloon | 576 (8.4) | 18 (6.3) |  |
| Intravenous GPI | 1049 (15.3) | 38 (13.3) | 0.408 |
| LVEF, % | 60.7 (7.7) | 60.0 (8.2) | 0.181 |
| Hemoglobin, g/L | 133.7 (15.5) | 130.2 (16.7) | <0.001 |
| Platelet, ×10^9^/L | 191.0 (157.0, 235.0) | 185.0 (159.0, 235.5) | 0.531 |
| Serum creatinine, μmol/L | 81.0 (70.0, 92.0) | 84.0 (72.0, 96.5) | 0.019 |
| Positive FOBT | 835 (12.2) | 35 (12.3) | 1.000 |
| Aspirin or indobufen |  |  | 0.410 |
| Aspirin | 6184 (90.3) | 262 (91.9) |  |
| Indobufen | 666 (9.7) | 23 (8.1) |  |
| P2Y_12_ receptor antagonist |  |  | 0.121 |
| Clopidogrel | 4564 (66.6) | 203 (71.2) |  |
| Ticagrelor | 2286 (33.4) | 82 (28.8) |  |
| Statin | 6750 (98.5) | 280 (98.2) | 0.878 |
| Ezetimibe | 970 (14.2) | 42 (14.7) | 0.852 |
| Fenofibrate | 87 (1.3) | 5 (1.8) | 0.658 |
| PCSK9i | 99 (1.4) | 5 (1.8) | 0.862 |
| RASI | 3754 (54.8) | 166 (58.2) | 0.278 |
| ARNI | 452 (6.6) | 23 (8.1) | 0.392 |
| Βeta-blocker | 4808 (70.2) | 202 (70.9) | 0.855 |
| Ivabradine | 78 (1.1) | 5 (1.8) | 0.504 |
| CCB | 2047 (29.9) | 86 (30.2) | 0.968 |
| Nitrate | 2232 (32.6) | 121 (42.5) | 0.001 |
| Diuretic | 514 (7.5) | 29 (10.2) | 0.120 |
| PPI | 2960 (43.2) | 128 (44.9) | 0.612 |

Values are shown as numbers (%), mean (standard deviation), or median (interquartile range).

Abbreviations: ACS = acute coronary syndrome; ARC-HBR = Academic Research Consortium-high bleeding risk; ARNI = angiotensin receptor-neprilysin inhibitor; ASD = absolute standardized difference; BMI = body mass index; CABG = coronary artery bypass grafting; CCB = calcium channel blocker; CKD = chronic kidney disease; DBP = diastolic blood pressure; FOBT = fecal occult blood test; GPI = glycoprotein Ⅱb/Ⅲa receptor inhibitor; LVEF = left ventricular ejection fraction; MI = myocardial infarction; NSTEMI = non-ST-segment elevation myocardial infarction; PCI = percutaneous coronary intervention; PCSK9i = proprotein convertase subtilisin/kexin type 9 inhibitor; PPI = proton pump inhibitor; PTCA = percutaneous transluminal coronary angioplasty; RASI = renin-angiotensin system inhibitor; SBP = systolic blood pressure; STEMI = ST-segment elevation myocardial infarction.


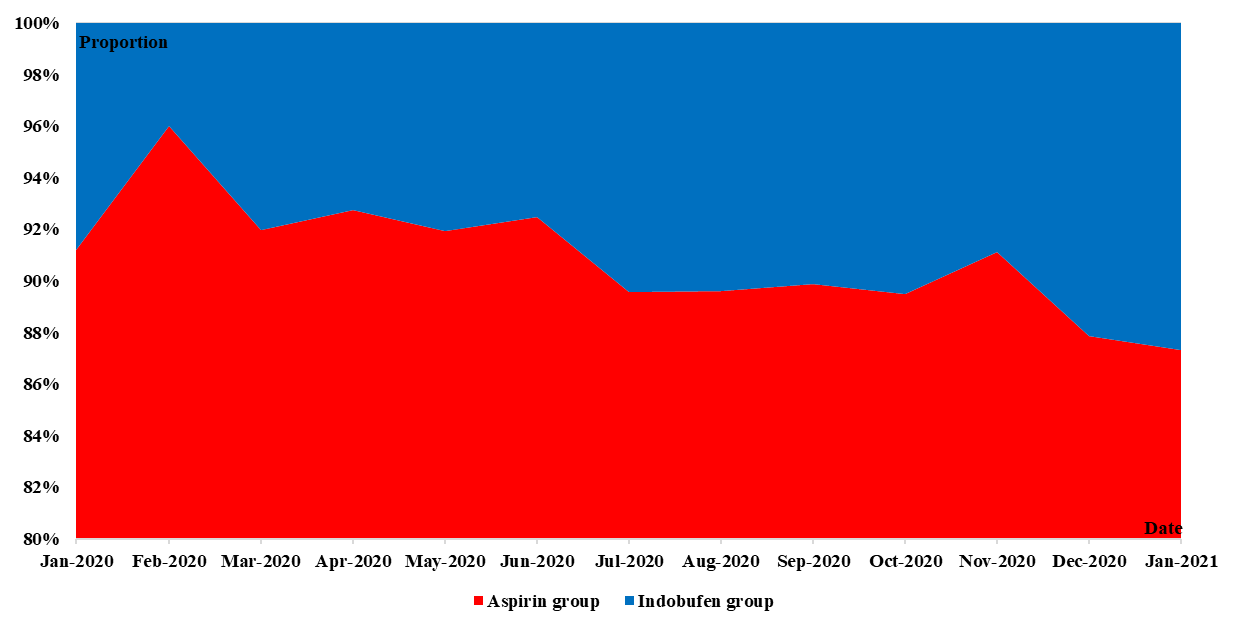
**Supplemental Figures**

**Figure S1**: Temporal trend of indobufen use.


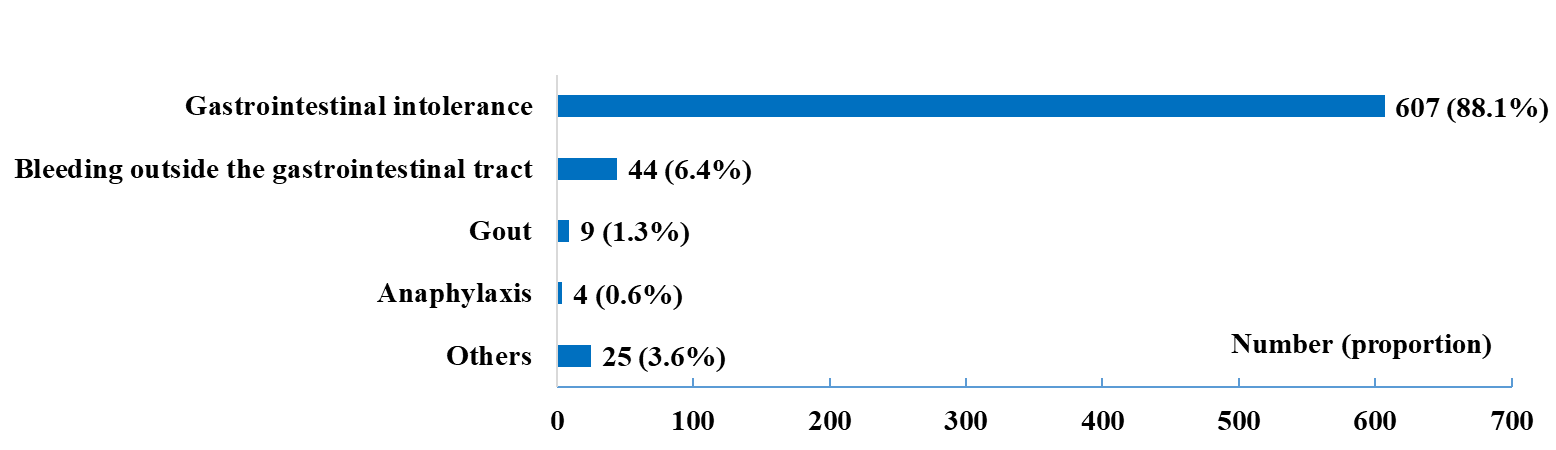
**Figure S2**: Manifestations of aspirin intolerance.

**Figure S3**: Comparison of ASD before and after matching.

The Love plot shows the change of ASD between the two groups before and after propensity score matching.

Abbreviations: ARC-HBR = Academic Research Consortium-high bleeding risk; ARNI = angiotensin receptor-neprilysin inhibitor; ASD = absolute standardized difference; BMI = body mass index; CABG = coronary artery bypass grafting; CCB = calcium channel blocker; CKD = chronic kidney disease; DBP = diastolic blood pressure; FOBT = fecal occult blood test; GPI = glycoprotein Ⅱb/Ⅲa receptor inhibitor; LVEF = left ventricular ejection fraction; MI = myocardial infarction; NSTEMI = non-ST-segment elevation myocardial infarction; PCI = percutaneous coronary intervention; PCSK9i = proprotein convertase subtilisin/kexin type 9 inhibitor; PPI = proton pump inhibitor; PTCA = percutaneous transluminal coronary angioplasty; RASI = renin-angiotensin system inhibitor; SBP = systolic blood pressure; STEMI = ST-segment elevation myocardial infarction.

**
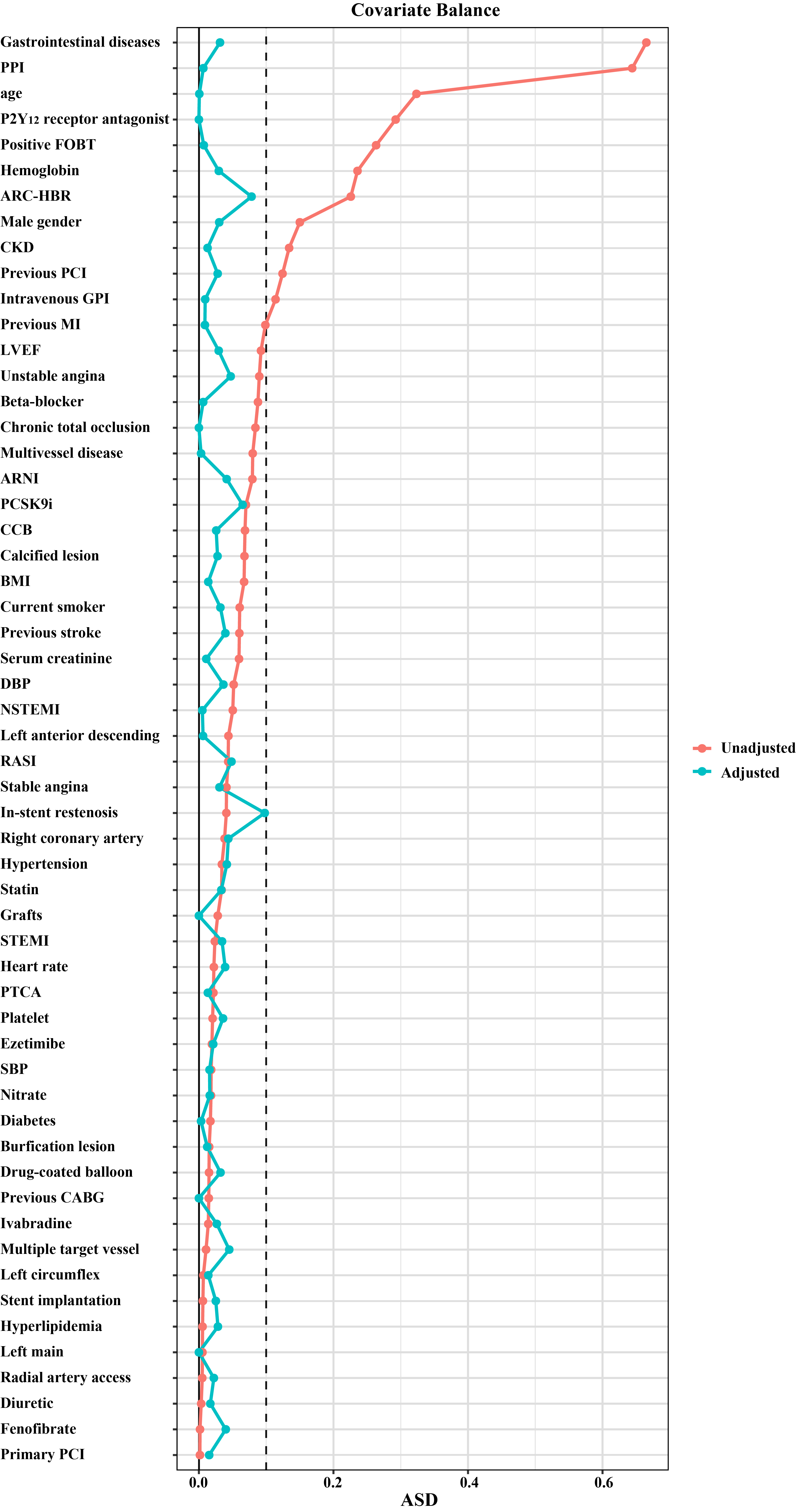
**

**Figure S4**: Distributions of propensity scores before and after matching.

The jitter plot (A) and histogram (B) show the propensity scores between the two groups before and after propensity score matching.

**
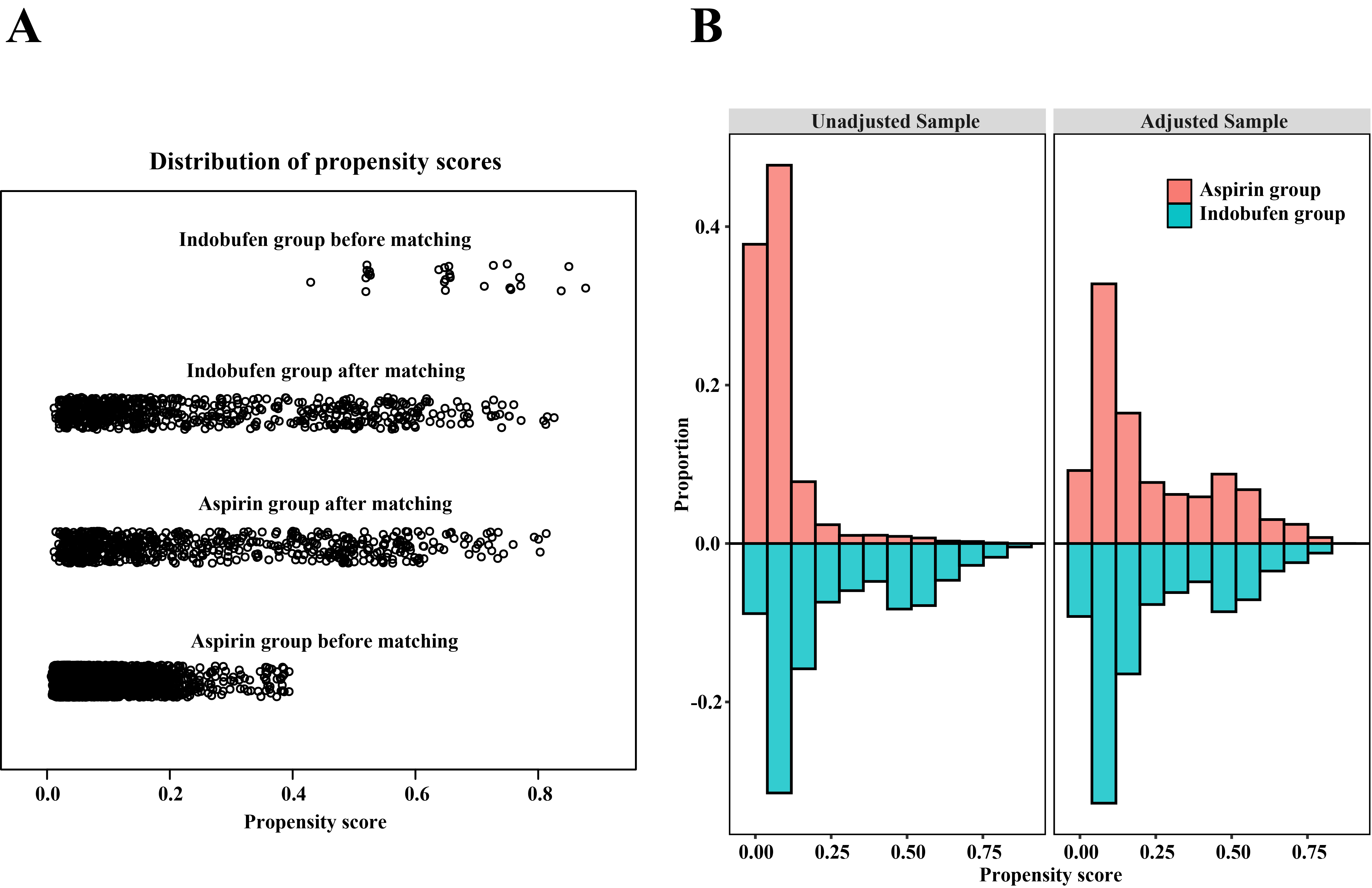
**

**Figure S5**: Sensitivity analysis.

It shows the sensitivity analysis for MACCE (A) and BARC type 2, 3, or 5 bleeding (B).

* Details of the adjusted covariates can be seen in **Supplemental Methods**.

Abbreviations: BARC = Bleeding Academic Research Consortium; CI = confidence interval; HR = hazard ratio; MACCE = major adverse cardiovascular and cerebrovascular events.


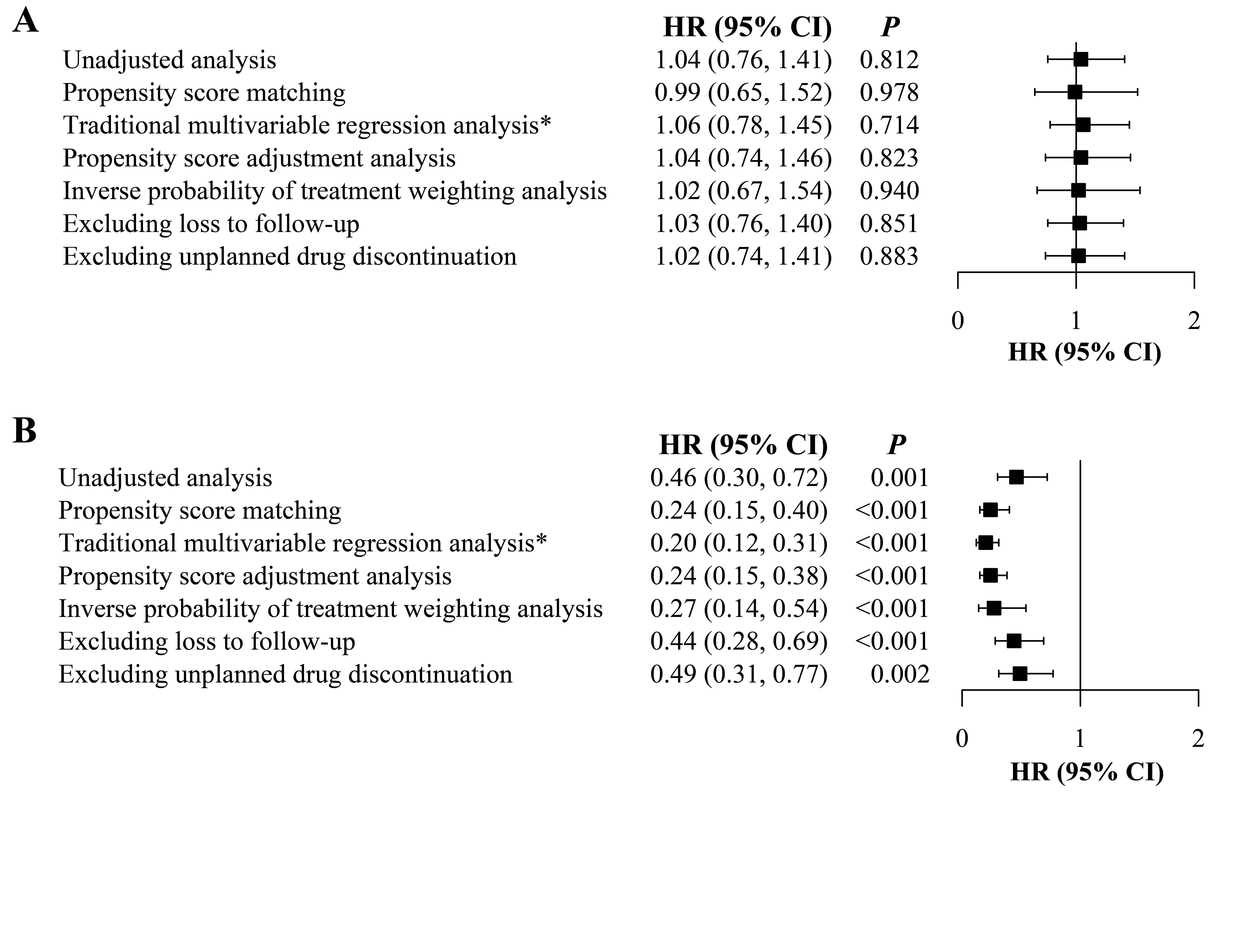


**Figure S6**: Reasons for unplanned drug discontinuation.

The percentile stacked bar plot shows the proportion of reasons for unplanned drug discontinuation in both groups during follow-up.

**
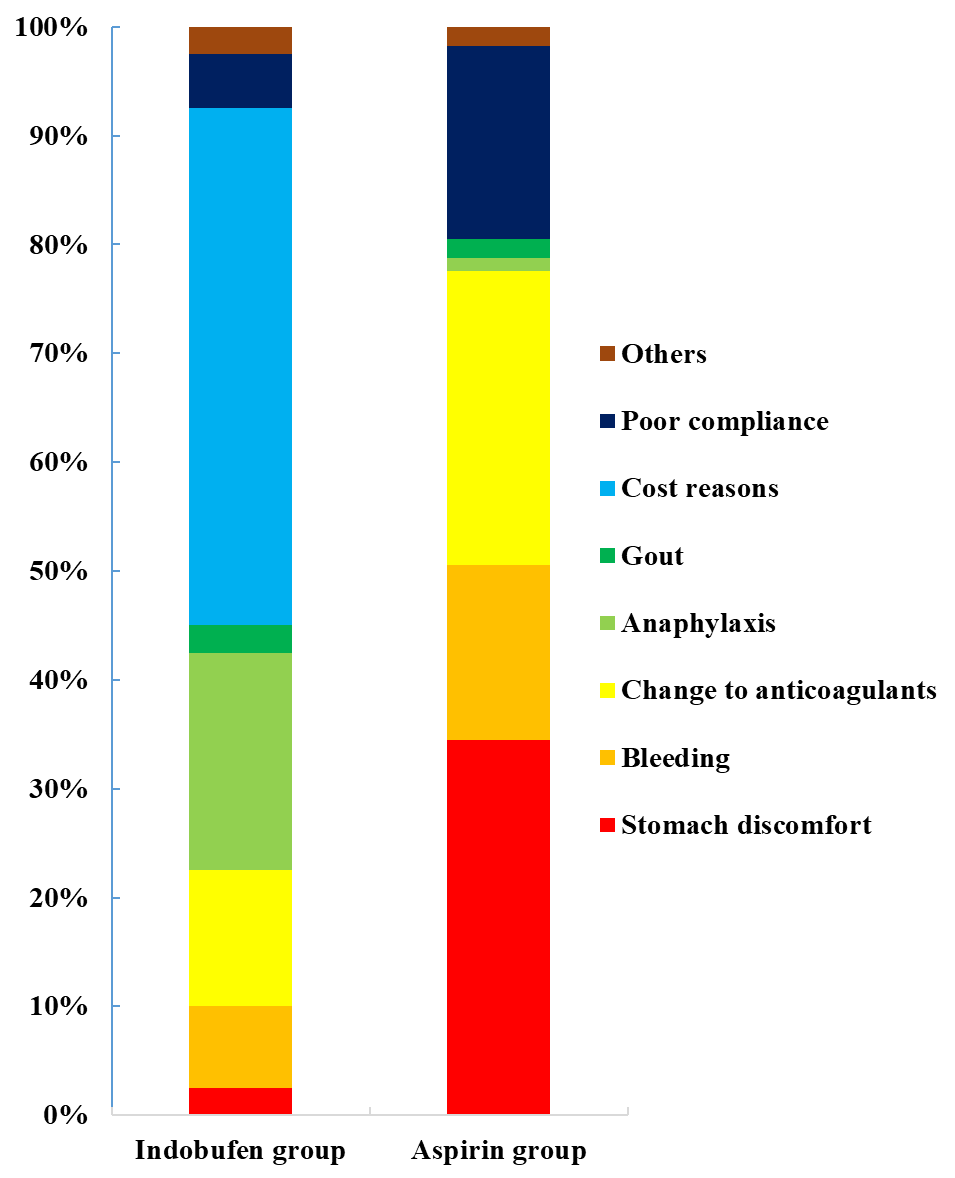
**

**STROBE Checklist**

Checklist of items that should be included in reports of ***cohort studies***

|  | Item No | Recommendation | Page No |
| --- | --- | --- | --- |
| **Title and abstract** | 1 | (*a*) Indicate the study’s design with a commonly used term in the title or the abstract | 1, 2 |
|  |  | (*b*) Provide in the abstract an informative and balanced summary of what was done and what was found | 2 |
| Introduction | | | |
| Background/rationale | 2 | Explain the scientific background and rationale for the investigation being reported | 3 |
| Objectives | 3 | State specific objectives, including any prespecified hypotheses | 3 |
| Methods | | | |
| Study design | 4 | Present key elements of study design early in the paper | 4 |
| Setting | 5 | Describe the setting, locations, and relevant dates, including periods of recruitment, exposure, follow-up, and data collection | 4-6 |
| Participants | 6 | (*a*) Give the eligibility criteria, and the sources and methods of selection of participants. Describe methods of follow-up | 4-6 |
|  |  | (*b*) For matched studies, give matching criteria and number of exposed and unexposed | 4-6 |
| Variables | 7 | Clearly define all outcomes, exposures, predictors, potential confounders, and effect modifiers. Give diagnostic criteria, if applicable | 4-6 |
| Data sources/ measurement | 8* | For each variable of interest, give sources of data and details of methods of assessment (measurement). Describe comparability of assessment methods if there is more than one group | 4-6 |
| Bias | 9 | Describe any efforts to address potential sources of bias | 4-6 |
| Study size | 10 | Explain how the study size was arrived at | NA |
| Quantitative variables | 11 | Explain how quantitative variables were handled in the analyses. If applicable, describe which groupings were chosen and why | NA |
| Statistical methods | 12 | (*a*) Describe all statistical methods, including those used to control for confounding | 6-7 |
|  |  | (*b*) Describe any methods used to examine subgroups and interactions | 7 |
|  |  | (*c*) Explain how missing data were addressed | 7 |
|  |  | (*d*) If applicable, explain how loss to follow-up was addressed | 6-7 |
|  |  | (*e*) Describe any sensitivity analyses | 7 |
| Results | | |  |
| Participants | 13* | (a) Report numbers of individuals at each stage of study—eg numbers potentially eligible, examined for eligibility, confirmed eligible, included in the study, completing follow-up, and analysed | 8 |
|  |  | (b) Give reasons for non-participation at each stage | 9-10 |
|  |  | (c) Consider use of a flow diagram | Fig. 1 |
| Descriptive data | 14* | (a) Give characteristics of study participants (eg demographic, clinical, social) and information on exposures and potential confounders | 8 |
|  |  | (b) Indicate number of participants with missing data for each variable of interest | 7 |
|  |  | (c) Summarise follow-up time (eg, average and total amount) | 8 |
| Outcome data | 15* | Report numbers of outcome events or summary measures over time | 8-9 |

| Main results | 16 | (*a*) Give unadjusted estimates and, if applicable, confounder-adjusted estimates and their precision (eg, 95% confidence interval). Make clear which confounders were adjusted for and why they were included | 8-9 |
| --- | --- | --- | --- |
|  |  | (*b*) Report category boundaries when continuous variables were categorized | NA |
|  |  | (*c*) If relevant, consider translating estimates of relative risk into absolute risk for a meaningful time period | NA |
| Other analyses | 17 | Report other analyses done—eg analyses of subgroups and interactions, and sensitivity analyses | 9-10 |
| Discussion | | | |
| Key results | 18 | Summarise key results with reference to study objectives | 10 |
| Limitations | 19 | Discuss limitations of the study, taking into account sources of potential bias or imprecision. Discuss both direction and magnitude of any potential bias | 14-15 |
| Interpretation | 20 | Give a cautious overall interpretation of results considering objectives, limitations, multiplicity of analyses, results from similar studies, and other relevant evidence | 10-14 |
| Generalisability | 21 | Discuss the generalisability (external validity) of the study results | 15 |
| Other information | | | |
| Funding | 22 | Give the source of funding and the role of the funders for the present study and, if applicable, for the original study on which the present article is based | 16 |

*Give information separately for exposed and unexposed groups.

**Note:** An Explanation and Elaboration article discusses each checklist item and gives methodological background and published examples of transparent reporting. The STROBE checklist is best used in conjunction with this article (freely available on the Web sites of PLoS Medicine at http://www.plosmedicine.org/, Annals of Internal Medicine at http://www.annals.org/, and Epidemiology at http://www.epidem.com/). Information on the STROBE Initiative is available at http://www.strobe-statement.org.
